# An Update on the Role of Serotonin and its Interplay with Dopamine for Reward

**DOI:** 10.3389/fnhum.2017.00484

**Published:** 2017-10-11

**Authors:** Adrian G. Fischer, Markus Ullsperger

**Affiliations:** ^1^Department of Neuropsychology, Institute of Psychology, Otto-von-Guericke University, Magdeburg, Germany; ^2^Center for Behavioral Brain Sciences, Magdeburg, Germany

**Keywords:** serotonin, 5-HT, dopamine, reward, optogenetics, drugs, co-release

## Abstract

The specific role of serotonin and its interplay with dopamine (DA) in adaptive, reward guided behavior as well as drug dependance, still remains elusive. Recently, novel methods allowed cell type specific anatomical, functional and interventional analyses of serotonergic and dopaminergic circuits, promising significant advancement in understanding their functional roles. Furthermore, it is increasingly recognized that co-release of neurotransmitters is functionally relevant, understanding of which is required in order to interpret results of pharmacological studies and their relationship to neural recordings. Here, we review recent animal studies employing such techniques with the aim to connect their results to effects observed in human pharmacological studies and subjective effects of drugs. It appears that the additive effect of serotonin and DA conveys significant reward related information and is subjectively highly euphorizing. Neither DA nor serotonin alone have such an effect. This coincides with optogenetically targeted recordings in mice, where the dopaminergic system codes reward prediction errors (PE), and the serotonergic system mainly unsigned PE. Overall, this pattern of results indicates that joint activity between both systems carries essential reward information and invites parallel investigation of both neurotransmitter systems.

## Introduction

Among the brain’s neuromodulators, serotonin (5-hydroxytryptamine, 5-HT) is likely the most ambivalent one with regard to its supposed importance for behavior and level of understanding. Serotonergic drugs are widely used in psychiatric disorders, abused as recreational drugs and liabilities in the serotonergic system have been identified as important etiological factors in many prevalent disorders. On the other hand, 5-HT is commonly described as “mysterious” in scientific contexts (Daw et al., 2002; Luo et al., 2015; Li et al., 2016), reflecting the fact that a unifying function of its physiological role has not been established and data are often inconclusive. Although an involvement of 5-HT in rewarding and aversive processing, hedonic experience, mood and higher cognitive functions such as consciousness or self reflection are undisputed, its precise contribution is controversial. These ambiguities reach deep into the history of studies on the serotonergic system. It has long been known from animal studies across different species that the raphe nuclei, the origin of most of the forebrain’s serotonergic innervation, are among the most potent areas inducing self stimulation equivalent to stimulation of the medial forebrain bundle or the ventral tegmental area (VTA; Miliaressis et al., 1975; Van Der Kooy et al., 1978; Rompre and Miliaressis, 1985). On the other hand, influential theories of 5-HT functioning position it as a behavioral inhibitor (Soubrié, 1986) essential in facilitating aversive processing (Tye et al., 1977), therefore, opposing the role ascribed to dopamine (DA; Deakin and Graeff, 1991; Daw et al., 2002; Cools et al., 2008a; Dayan and Huys, 2009). These partly contradictory functions may be explained by interactions between different cell types within the raphe that depend on serotonin.

Here, we will outline the role of different cell types in raphe nuclei and the cell-type specific anatomy of the serotonergic system. We first review recent studies on serotonin’s role in human and animal behavior related to rewarding and aversive processing. We then relate these findings to novel results in which serotonergic and non-serotonergic signals including co-release of other transmitters related to the 5-HT system have been investigated via genetic targeting approaches. We thereafter focus on self-stimulation studies as a measure of reward in animals, that offers the possibility to relate findings to human behavior including subjectively experienced reward induced by drugs. In conclusion, we will summarize the importance of considering different cells and the interplay of neuromodulatory systems and neurotransmitter co-release when discussing the role of neuromodulators like 5-HT and DA.

## The Serotonergic System

The density of 5-HT receptors in the cortex shows a descending rostro-caudal gradient, indicative of an especially prominent involvement of 5-HT in higher cognitive functions (Kranz et al., 2010; Celada et al., 2013). Serotonergic afferents are provided by only a small group of cells located in the raphe area of the midbrain. Two major nuclei here consist in the dorsal and median raphe (DRN, MRN) which comprise around 160,000 and 60,000 serotonergic neurons in humans, respectively (Charnay and Léger, 2010). The majority of serotonergic input to the forebrain is provided by the DRN, on which we focus here. There are considerable differences in the percentage of serotonergic neurons within the DRN across species: in humans, 70% of DRN neurons were found to contain 5-HT (Baker et al., 1991), whereas in cats (Léger and Wiklund, 1982) and rats, serotonergic neurons do not constitute the majority of the cell population in the DRN. Of the remaining cells, glutamatergic and GABAergic neurons constitute the majority (Hornung, 2003; Bang and Commons, 2012) and the GABAergic cells mediate inhibitory feedback within the DRN (Liu et al., 2000). Aside from cell type diversity, co-release of neurotransmitters is increasingly recognized as functionally relevant (El Mestikawy et al., 2011). Neurons that express tryptophan hydroxylase 2 (TpH2), the rate-limiting enzyme in 5-HT synthesis that is almost exclusively expressed in serotonergic neurons, have been found to express the vesicular glutamate transporter type 3 (VGluT3) which transports glutamate into presynaptic vesicles in non-primarily glutamatergic neurons (Hioki et al., 2010). Glutamate release from these 5-HT neurons has been repeatedly observed (Johnson, 1994; Liu et al., 2014; Qi et al., 2014). Co-release is by no means restricted to the serotonergic system, but has been acknowledged in dopaminergic neurotransmission as well (Stuber et al., 2010; Tecuapetla et al., 2010). This intrinsic interlacing between neurotransmitter systems suggests that the functional relevance of neuromodulatory systems is difficult to judge when dissecting individual components of a physiological system consistently releasing more than a single neurotransmitter.

A major goal in understanding neuromodulatory systems is to combine correlational evidence, for example from imaging studies (Macoveanu, 2014), with pharmacological interventions, and provide a plausible picture bridging results from direct recordings of specific activation or lesion studies in animals and pharmacological interventions, eventually consistent with self reports in humans. Methods that affect overall serotonergic tone are dietary acute tryptophan depletion (ATD; Fadda et al., 2000) which reduces 5-HT, tryptophan loading which presumably increases 5-HT (Young, 1996), and administration of SSRI which increase 5-HT levels, but could decrease co-release of glutamate (Fischer et al., 2015c). Direct agonists to 5-HT receptors have been used in various studies with complex results, likely due to the diversity of 5-HT receptors (Hayes and Greenshaw, 2011). A major expectation of novel optogenetic methods, that allow to specifically target, record, activate and inhibit monoaminergic neurons, and to dissociate them, for example from inhibitory GABAergic neurons, is to provide evidence for 5-HT’s major physiological function which then could be used to reconcile results of pharmacological studies.

## Afferents of the Serotonergic System

Given the relevance of serotonergic neuromodulation for the development of anxiety or depression (Caspi et al., 2003; Sachs et al., 2015), control of serotonergic signaling is highly important. It has only recently become possible to specifically trace inputs to identified serotonergic neurons, differentiating them, for example, from afferent neurons that synapse onto GABAergic cells. This can be done by specifically targeting a tracer to genetically defined cell populations (such as 5-HT neurons) that express a protein required for the tracer to travel retrogradely under a specific promoter active in the cell population. When targeting, for example, the serotonin transporter promoter gene, this technique provides a means to specifically localize monosynaptic inputs to 5-HT neurons in mice. Studies using this technique found that multiple brain regions project monosynaptically to DRN 5-HT neurons (Ogawa et al., 2014; Pollak Dorocic et al., 2014), demonstrating the high level of control exerted over the serotonergic system. Input to defined serotonergic DRN, but not MRN, and defined dopaminergic VTA neurons is quantitatively and hodologically similar (Watabe-Uchida et al., 2012; Ogawa et al., 2014), compatible with strongly complementary functions in both systems. Among these input regions are the PFC and the lateral habenula (LHb), which both provide mainly excitatory input to serotonergic as well as GABAergic neurons in the DRN (Pollak Dorocic et al., 2014; Weissbourd et al., 2014; Geddes et al., 2016; Zhou et al., 2017). The net effect of such an input may be excitatory or mediate feed-forward inhibition. Additionally, another major input pathway to the raphe nuclei includes a mainly GABAergic relay of DRN inputs from LHb via the rostromedial tegmental nucleus (Jhou et al., 2009; Sego et al., 2014).

Inputs from the PFC can modulate and shift the degree of inhibition or excitation in the DRN (Geddes et al., 2016). Functionally, stimulation of PFC neurons projecting to the DRN was found to influence motivation: stimulation of this pathway increased, whereas silencing decreased motivation in rats in the forced-swim task (Warden et al., 2013). Subcortical areas send both excitatory and inhibitory projections onto serotonergic neurons, constituting a push-pull regulatory mechanism (Zhou et al., 2017). The LHb has been suggested to mediate aversive signals transmitted to the dopaminergic and serotonergic system (Matsumoto and Hikosaka, 2007; Hikosaka, 2010). Electrical stimulation of the LHb inhibits DRN cell firing (Wang and Aghajanian, 1977) and LHb lesions increase DRN 5-HT levels (Yang et al., 2008) in rats. This up-stream input to the DRN could be integrated by GABAergic neurons that control serotonergic neuronal activity. Functional relevance of this interplay between GABAergic and serotonergic DRN neurons has been demonstrated for the expression of avoidance following social defeat stress in mice. Following repeated exposure to dominant conspecifics, GABAergic neurons in the DRN increased in excitability which led to decreased 5-HT activity (Challis et al., 2013). Optogenetic silencing of GABAergic DRN neurons prevented expression of behavioral avoidance, indicative of a causative influence of GABAergic DRN neurons on the development of stress-related avoidance behavior. Furthermore, pharmacogenetically specific inactivation of LHb ameliorated the consequences of social defeat stress in an antidepressant like fashion even in mice in which SSRI were not effective due to a lack of 5-HT synthesis (Sachs et al., 2015), compatible with the idea that the LHb DRN pathway exerts strong control over DRN signaling where 5-HT itself is only one part of the whole picture. However, an additional debate exists as to the actual valence of serotonergic signaling along the reward axis.

## Valence and Serotonin

An influential hypothesis holds that 5-HT controls behavioral inhibition in an aversive context (Soubrié, 1986) such as punishment, monetary losses or omitted rewards, thus ascribing opposite functions to DA and 5-HT (Daw et al., 2002). Vice versa, many studies have shown that 5-HT influences processing of rewards (Kranz et al., 2010), suggesting rather complementary roles for both systems.

Overall, pharmacological manipulations of serotonergic neurotransmission in humans provided striking evidence for an involvement of 5-HT in punishment processing (Evers et al., 2005; Chamberlain et al., 2006; Crockett et al., 2009; Geurts et al., 2013; Macoveanu et al., 2013). Dietary lowering of serotonergic activity abolished reaction slowing induced by punishments (Crockett et al., 2012), whereas acute SSRI administration as well as genetically determined higher 5-HT levels are associated with increased reaction slowing following errors (Fischer et al., 2015b). Similarly, ATD disturbed the association of past actions with punishments, but not rewards (Tanaka et al., 2009), which would suggest that 5-HT levels positively covary with the ability to memorize and utilize aversive events. However, ATD increased participants’ ability to predict negative outcomes of observed choices (Cools et al., 2008b; Robinson et al., 2012) and increased the BOLD response to errors in fMRI in the pre-frontal cortex (Evers et al., 2005). On the other hand, the suggestion that 5-HT mediates reward processing (Kranz et al., 2010; Luo et al., 2016) has likewise received considerable empirical support (Rogers et al., 2002; Cools et al., 2005; Del-Ben et al., 2005; Roiser et al., 2006; Tanaka et al., 2007; Seymour et al., 2012), while other studies support both punishment as well as reward processing (McCabe et al., 2010; Palminteri et al., 2012; Worbe et al., 2016; Scholl et al., 2017). Thus, manipulations of serotonergic tone affect reward and punishment processing, but the net effect, an impairment or facilitation, is not entirely consistent across studies.

Comparable to human studies, animal research has demonstrated involvement of the serotonergic system in processing both punishments and rewards. Using a reversal learning task with both rewards (juice) and punishments (noise) in marmosets, Rygula et al. (2015) found that local depletion of 5-HT in the amygdala or frontal cortex reduced overall feedback sensitivity, independent of valence. Similarly, rodent studies found that the overall effect of 5-HT manipulations can affect punishment or reward sensitivity, depending on method (SSRI, ATD, 5-HT depletion), dosage and duration of treatment (Bari et al., 2010). SSRI and genetically increased 5-HT levels were found to reduce appetitive operant responses (Sanders et al., 2007) but increased win-stay behavior during reversal learning in mice (Brown et al., 2012). Strong depletion of 5-HT decreased instrumental reward-based reinforcement-learning in rats (Izquierdo et al., 2012). Similarly, 5-HT depletion in the frontal cortex of marmosets disrupted acquisition of responding to appetitive conditioned reinforcement but not extinction (Walker et al., 2009). Akin to human studies, overall manipulations of serotonergic tone in animals sometimes affect reward and sometimes punishment processing. Overall, this suggests the need to extend the scope of one neuromodulatory system to its anatomical and neurochemical context.

## Is Serotonin or DRN Activity Rewarding?

The DRN has since long been identified as one of the primary brain areas that promote self stimulation that strongly reinforces behavior via the dopaminergic system (Rompre and Miliaressis, 1985). However, it was until recently unclear if this mechanism actually relied upon cells originating within the DRN or if stimulation activated passing fibers, as well as if mediating cells are truly serotonergic. The structural connectivity between VTA and DRN has recently been described considerably more precisely by novel cell-type specific tracing techniques. It was found that dopaminergic VTA neurons in mice receive both the densest projections, as well as the second most numerous, after striatal projections, from the DRN (Watabe-Uchida et al., 2012). This projection was found to be mainly glutamatergic, but additionally contains 5-HT co-releasing neurons (McDevitt et al., 2014; Qi et al., 2014). This pathway drives self-stimulation and conditioned place preference via asymmetrical synapses to mesostriatal VTA DA neurons (Qi et al., 2014). Both blockade of striatal D1 receptors as well as blockade of glutamatergic signals in the VTA, abolishes these effects. Additionally, inhibitory GABAergic projections from VTA and striatum regulate 5-HT activity, forming a feedback-loop that intertwines DA and 5-HT systems in the reward circuitry (Pollak Dorocic et al., 2014), although tracing studies suggest that 5-HT is in a stronger position to control DA activity than vice versa (Ogawa et al., 2014).

Single-neuron recording studies suggested that DRN neurons, that putatively contain 5-HT, process reward. In rats and monkeys, these DRN neurons were found to reflect the magnitude of delivered liquid rewards as well as overall reward likelihood (Nakamura et al., 2008; Bromberg-Martin et al., 2010; Inaba et al., 2013). Using calcium imaging and fiber photometry, a method to observe activity in specifically targeted neuronal populations, it was recently shown that serotonin transporter positive (SERT^+^) DRN 5-HT neurons displayed strong reward- but not aversion-related activity modulations in freely behaving mice (Li et al., 2016). These neurons increased activity during consumption of primary reinforcers (sugar, sex), and while animals waited for delivery of rewards. This confirms a series of previous experiments which suggested 5-HT to mediate impulse control whilst anticipating a future reward (Miyazaki K. W. et al., 2012; Miyazaki K. et al., 2012; Miyazaki et al., 2014), and speaks towards involvement of SERT^+^ 5-HT neurons in primary reward processing. Interestingly, activity of putative (Inaba et al., 2013; Hayashi et al., 2015) and optogenetically defined 5-HT neurons display value-related signals on different time scales (Cohen et al., 2015). In head-restrained mice, Cohen et al. (2015) found that serotonergic neurons display very brief responses to rewards and punishments, but sustained activity possibly reflecting the “beneficialness” (Luo et al., 2016) of the current environment, or the motivational state, for periods of up to 10 s (Cohen et al., 2015). These long-lasting changes are reflected in rather low changes in firing rates in the range of 1–2 spikes/s, which nonetheless might exert significant influences in serotonergic innervation on target structures especially given the low baseline firing rate observed in most 5-HT neurons.

Optogenetic stimulation provided new insights in the functional effects of activation of specific DRN neurons. Liu et al. (2014) expressed a light sensitive ion channel in DRN neurons targeted via an enhancer region coupled to the Pet-1 gene, which is selectively (yet not exclusively) expressed in serotonergic neurons (Scott et al., 2005). Stimulation of Pet-1^+^ neurons was found to be highly rewarding, inducing conditioned place preference outlasting the stimulation period, instrumental learning and favorable competition against a natural reinforcer (Liu et al., 2014). These effects mostly depended on co-release of glutamate and were reduced, yet not absent, in mice lacking VGluT3. Consistently, direct targeting of VGluT3^+^ neurons projecting from DRN to VTA, out of which only some contain 5-HT, drove vigorous self stimulation (Qi et al., 2014), indicating that DRN neurons can induce burst firing in VTA DA neurons which drives behavioral reinforcement (Grace et al., 2007). Another study found that orbitofrontal cortex neurons whose activity reflected prospective natural reinforcers also coded the prospective intensity of Pet-1^+^ DRN stimulation (Zhou et al., 2015), indicating similarity between natural reinforcers and artificial DRN stimulation. However, a very similar stimulation protocol again targeting Pet-1^+^ DRN neurons did not replicate the rewarding effects reported before (McDevitt et al., 2014).

Another targeting approach for 5-HT neurons within the DRN for optogenetic activation, is genetic tagging of the SERT. Targeting SERT is hypothesized to reduce overlap with glutamatergic populations (Luo et al., 2015), yet additional empirical validation for this assumption is needed. As a caveat, targeting 5-HT neurons via SERT requires heterozygous knock-in, effectively de-activating one SERT allele, which in itself alters serotonergic activity (Mathews et al., 2004). In this line of research, rewarding effects of DRN 5-HT neuron stimulation could not be replicated. Stimulation was neither found to induce sustained place preference, nor reinforce behavior (Fonseca et al., 2015; Correia et al., 2017). Additionally, when 5-HT neurons were targeted via the regulatory elements of the rate-limiting enzyme in 5-HT synthesis, TpH2, no directly rewarding effects of stimulation were observed (Miyazaki et al., 2014). Recently, Correia et al. (2017) found that short-term activation of SERT^+^ DRN neurons led to behavioral slowing again in the absence of rewarding effects, which however did not interfere with motivated behavior.

Another core feature that ties reward processing to learning and behavioral adaptation is the computation of teaching signals in the form of reward or punishment prediction errors (PE), which has been ascribed as a central function of the DA system. Recently, it was found that different subpopulations of dopaminergic neurons with specific projection regions respond differentially to reward, novelty and aversiveness. Projection neurons to the ventral striatum reflect reward, but appear insensitive to aversive events and novelty (Eshel et al., 2016; Menegas et al., 2017), which additionally can be modulated by reward context (Matsumoto et al., 2016). On the contrary, other VTA neurons (Matsumoto and Hikosaka, 2009), as well as defined dopaminergic neurons (Cohen et al., 2012), reflect aversive stimuli and novelty, but these seem to project to more caudal striatal regions (Menegas et al., 2017). Some recent studies investigated coding of reward PEs in defined serotonergic neurons. Matias et al. (2017) reported that SERT^+^ 5-HT neurons in DRN on population level reflect positive reward PEs, consistent with several other studies (Nakamura et al., 2008; Inaba et al., 2013; Cohen et al., 2015; Hayashi et al., 2015) and very similar to DA neurons in VTA. However, while reward PE coding DA neurons decreased firing rates when an expected reward was omitted or replaced with likely aversive events (an air puff targeted to the eye), constituting a signed PE signal, 5-HT neurons reflected mainly unsigned PEs, or surprise. Interestingly, 5-HT neurons were slower to adapt to expectancy changes, causing a shift in putative relative availability of 5-HT and DA during the learning of reward expectations. This leads to a situation in which, for example in reversal learning tasks, a switch from a previously good to a now bad stimulus is accompanied by an early decline in DA activity, but a longer lasting 5-HT signal that only later on adapts to the new expected level of reward. This may explain why 5-HT has in many cases been ascribed a mainly aversive role opponent to DA (Daw et al., 2002). It remains an open question if GABAergic neurons in DRN represent expected value, similar to the role of GABAergic neurons in VTA in dopaminergic reward PE calculation (Cohen et al., 2012; Eshel et al., 2015).

An interesting observation is that optogenetically defined serotonergic neurons were found to respond earlier, although less vigorously, to reward predicting cues than DA neurons (Cohen et al., 2015), yet later than some cortical reward related signals (Ullsperger et al., 2014). This may indicate that DRN reward related activity represents a high level modulatory control of the brain’s reward system, depending on input from upstream cortical areas involved in reward processing.

Overall, it appears that serotonergic neurons code reward signals consistent across different species, yet their optogenetic activation is only rewarding when either glutamate is co-released or glutamatergic DRN neurons are targeted. These glutamatergic cells project strongly to VTA and are able to recruit the dopaminergic reward circuitry. Aversive events are not consistently (Li et al., 2016) coded by serotonergic neurons in animal studies. This may be explained by the fact that in most of these studies animals were head-restrained (Cohen, 2015; Matias et al., 2017). Restrainment itself might be a stressor interfering with physiological neuronal activity patterns of a neural modulator thought to be involved in reward processing and mood.

## The Role of 5-HT in Drug-Induced Euphoria and Controlling Reward Seeking Behavior

Low levels of 5-HT are a known diathesis for the development of anxiety disorders, depression (Jacobsen et al., 2012; Sachs et al., 2015), but also drug addiction (Ducci and Goldman, 2012). Additionally, most addictive drugs either cause increased 5-HT release or directly agonize 5-HT receptors. Two essential components of reward can be defined as “liking”, reflecting a pleasurable subjective state, and “wanting”, reflecting the reinforcing properties of a situation or substance (Berridge et al., 2009). Assessing liking in animal models mostly relies on facial expression studies, for example in mice, where it was demonstrated that DA, while reinforcing behavior, does not induce liking (Wyvell and Berridge, 2000; Leyton et al., 2002; Berridge and Robinson, 2003). On the other hand, subjective drug effects reported in humans can be informative about underlying neurotransmitter systems and how they mediate effects.

The group of monoamine releasers, which increase DA, 5-HT and norepinephrine (NE), and their most commonly abused variants such as cocaine, amphetamine and Methylenedioxymethamphetamine (MDMA or ecstasy) are, after cannabis, the most widely abused illegal drugs^1^. Contrary to initial assumptions, potency to release NE does not seem to significantly alter addictive potency of these substances (Banks et al., 2014), but the relative potential to release 5-HT over DA has been found to affect both a drug’s euphoric, as well as addictive properties. Interestingly, the potency of drugs to release 5-HT, even when effects on DA and NE release are comparable, negatively correlates with its potency as a behavioral reinforcer (Wee et al., 2005). This was demonstrated using various amphetamine derivates in monkeys with comparable NE- and DA-, yet different 5-HT-releasing properties. Furthermore, individual monkeys with more pronounced 5-HT release induced by MDMA consistently self-administered the drug less than individuals with lower release. Addition of the serotonin releasing agent fenfluramine to self-administered amphetamine also decreased self-administration (Wee and Woolverton, 2006). Consistently, destruction of the serotonergic system using the selective neurotoxin 5,7-DHT increased MDMA self-administration rate and acquisition speed in rats (Bradbury et al., 2013). This suggests that 5-HT balances the behaviorally reinforcing effects of DA.

Another way to assess behavioral control over reward behavior in laboratory settings is to combine intracranial self stimulation (ICSS) of rewarding areas (often the medium forebrain bundle) with administration of drugs. DA releasing agents have been shown in this setting to increase self stimulation, which is interpreted as a context dependent facilitation of addictive behavior. Interestingly, selective 5-HT releasers inhibit ICSS (Olds and Yuwiler, 1992), and moreover the relative specificity to release 5-HT compared with DA negatively covaries with ICSS facilitation (Bauer et al., 2013). However, this general inhibitory influence of 5-HT over ICSS is simplified, and other studies indicate that the overall effect of 5-HT on brain stimulation depends, among other factors, on the locus of 5-HT application (Kranz et al., 2010). Although addictive behavior evolves over a longer time scale and includes complex adaptations in the serotonergic system (Müller and Homberg, 2014), there is evidence from animal studies suggesting that integrity of the serotonergic system and higher 5-HT release, reduces drug self-administration.

Human studies that combine pharmacological manipulations of the 5-HT system and imaging techniques, have not yielded an unambiguous picture (Macoveanu, 2014), in part due to the difficulty to capture brainstem signals related to DRN or MRN activity. Tanaka et al. (2004) found increased activity overlapping with the DRN, when participants had to endure short-term losses to obtain long-term rewards, akin to effects observed in the dorsal striatum. This striatal signal was modulated by manipulations of 5-HT levels in human participants, and the covariation between dorsal striatum and long-term rewards was positively dependent on 5-HT levels (Tanaka et al., 2007). Furthermore, ATD in humans was found to reduce the impact of previous rewards on current choices, and increased the tendency to repeat previous choices in a dynamically changing reinforcement learning task (Seymour et al., 2012).

These findings translate to human abuse behavior and also appear to dissect a drug’s rewarding properties, mapping wanting to DA and speculatively liking to 5-HT, in an additive manner. Epidemiological data suggest that MDMA has a significantly lower abuse rate compared to monoamine releasers with less serotonergic action, like amphetamines (Degenhardt et al., 2010). On the other hand, even compared to high doses of amphetamine, MDMA is subjectively experienced as more pleasurable, positive mood inducing and euphorizing (Camí et al., 2000; Tancer and Johanson, 2003; Carhart-Harris et al., 2015). More selective 5-HT releasers that spare DA, surprisingly, are neither reinforcing (Woods and Tessel, 1974), nor experienced as pleasurable by humans. In fact, the selective 5-HT releaser m-chlorophenylpiperazine (mCPP) was found to reduce positive mood and euphoria (Tancer and Johanson, 2003), and acute intravenous administration of an SSRI in healthy participants, increased self rated sadness and incompetence (Fischer et al., 2015a). This suggests that 5-HT under physiological circumstances balances reinforcing effects of DA while itself being neither reinforcing nor rewarding, yet may be essential to inhibit reinforced behavior in order to promote behavioral flexibility (Branchi, 2011; Fischer et al., 2015b; Matias et al., 2017).

In short, consistent with subjective drug effects, positive reward may be reflected by a combination of DA and 5-HT, whereas punishment could be reflected by 5-HT and absence, or reduction, of DA (Cohen et al., 2015).

## Methodological Considerations

We put effort in this review to include especially findings from studies employing recent state-of-the art methods, such as optogenetics. These promised to resolve many contradictions regarding the 5-HT system by unequivocally identifying and interfering with specific neural populations, for which thus far identification was relatively unspecific using mostly either firing patterns or receptor dependent changes in activity. However, even these specific methods yield contradictory results. For example, doubtlessly direct DRN stimulation is rewarding, as known from self stimulation studies (Miliaressis et al., 1975; Van Der Kooy et al., 1978; Rompre and Miliaressis, 1985), and confirmed by optogenetic stimulation of either mostly glutamatergic non-serotonergic DRN output neurons (McDevitt et al., 2014) or 5-HT and glutamate co-releasing neurons (Liu et al., 2014; Qi et al., 2014). A more 5-HT specific stimulation profile, targeting SERT^+^ or TpH2^+^ neurons, on the other hand, does not reproduce rewarding effects (Miyazaki et al., 2014; Fonseca et al., 2015; Correia et al., 2017), possibly due to a lack of glutamatergic signaling, and acute SSRI administration is rarely reported as rewarding (Fischer et al., 2015a). An interpretation of this absence of effects appears complicated if such stimulation differs from physiological activity. It is currently unknown whether physiological inputs from specific regions to the DRN asymmetrically synapse onto purely serotonergic, glutamatergic, co-releasing, or other, e.g., GABAergic neurons. Thus, optogenetic stimulation may in some cases be over-specific in the sense of evoking an unphysiological signal that may not induce the same behavioral effects as normally occurring activation of serotonergic pathways by stimulating neurons that would usually fire independently.

On the other hand, it is well established that the group of raphe nuclei is involved in different physiological functions depending on their topography (Hale and Lowry, 2011). Moreover, distinct afferent projection profiles have been demonstrated. While PFC and LHb project bilaterally to the DRN, other regions such as amygdala and hypothalamus, asymmetrically synapse to ipsilateral parts (Zhou et al., 2017). Recently, it was found that optogenetic stimulation of SERT^+^ DRN projection neurons to the bed nucleus of the stria terminalis induces fear and anxiety (Marcinkiewcz et al., 2016), whereas the same stimulation of SERT^+^ neurons in the DRN itself, does not display this effect (Correia et al., 2017). This raises the question whether: (a) isolated stimulation of single pathways may be over-specific; or (b) activation of neuronal populations within a region may still not be specific enough.

Furthermore, it is crucial to incorporate the possibility of co-release of other neurotransmitters from monoaminergic neurons, which can lead to very different effects when comparing drug studies, e.g., employing agonists or releasers, which circumvent co-release, with stimulation studies, which induce co-release depending on the exact method used to target specific neurons (Hu, 2016). Considering such co-release-dependent discrepancies may explain puzzling effects of drugs, which often do not align with physiological hypotheses (Fischer et al., 2015c).

## Conclusion

Afferent and efferent projections, hodological properties, the time course of individual neural activity bridging short latency and longer lasting activity modulations, all position the serotonergic system ideally to extract motivationally salient information and induce focused attention to guide goal oriented actions towards rewards. Human and animal studies that manipulated 5-HT levels strongly support serotonin’s role in affective processing, but the direction of association and specificity for aversive or rewarding events remains controversial. Optogenetic studies in rodents suggest that DA and 5-HT jointly guide learning by positive coding of reward PE signals, whereas absence of DA and presence of 5-HT is associated with reward omission, and possibly punishment PE signals. Furthermore, the DRN controls release of 5-HT to many projection regions and can recruit dopaminergic reward circuits via glutamate. Activation of purely serotonergic cells was found to be mostly non-rewarding or purely inhibitory. Taken together, this could be interpreted such that manipulations increasing 5-HT signals without a consecutive DA increase, mimics aversive events. On the other hand, direct DRN stimulation, specific stimulation of glutamate-releasing and co-releasing DRN cells, induces rewarding effects comparable to direct VTA stimulation (McDevitt et al., 2014). It therefore appears that specific DRN neuronal populations control DA-related reward behavior via glutamate release and co-release. Thus, reward appears most likely mediated via glutamatergic excitation of DA neurons, at least in part, by input from DRN (Liu et al., 2014; McDevitt et al., 2014; Qi et al., 2014) and parallel release of 5-HT. In accordance with this, subjective self-reports of human subjects suggest that a singular increase in 5-HT or DA release is not experienced as pleasurable, but the conjoint increase is highly pleasurable. An interesting additional possibility is that 5-HT neurons may furthermore integrate DA effects over time into longer-lasting affective signals conveyed in a tonic fashion (Cohen et al., 2015). This is compatible with the idea that 5-HT encodes beneficialness (Luo et al., 2016), and may signal motivation to either maintain or switch current behavior, for example displayed by 5-HT’s role in facilitating patience for future rewards (Miyazaki et al., 2014). Combined with the finding that 5-HT levels via immediate release or pretreatment bivalently modulate the potency of highly addictive substances such as amphetamines or cocaine (Wee et al., 2005; Cunningham and Anastasio, 2014), it may be that it functionally orchestrates the brains reward systems via parallel fast activation and slow longer-lasting inhibition. Thus, 5-HT additionally appears to control motivationally rewarding effects of DA as evidenced via drug addiction.

Highly specific methods to stimulate neuromodulatory systems reveal the complexity and entanglement between neuromodulatory systems on multiple levels, but do not provide simple answers. As more data is collected, it appears that judging the effect of one neuromodulatory system alone is unphysiological. However, converging between pharmacological effects in humans and optogenetic stimulation in animals, DA and 5-HT combined provide signals that interdependently are sufficient to guide reward related behavioral adaptations, and induce subjective reward. This complexity has direct consequences for the interpretation of pharmacological, genetic, and correlational studies in humans and indicates that DA and 5-HT do not have opposing functions (Daw et al., 2002), but rather could in concert provide a combined reward signal, whereas its dissociation may encode punishment.

## Author Contributions

AGF wrote the manuscript, AGF and MU discussed, corrected and conceived the manuscript.

## Conflict of Interest Statement

The authors declare that the research was conducted in the absence of any commercial or financial relationships that could be construed as a potential conflict of interest.

## Footnotes

^1^www.globaldrugsurvey.com
